# Is Impact of Statin Therapy on All-Cause Mortality Different in HIV-Infected Individuals Compared to General Population? Results from the FHDH-ANRS CO4 Cohort

**DOI:** 10.1371/journal.pone.0133358

**Published:** 2015-07-22

**Authors:** Sylvie Lang, Jean-Marc Lacombe, Murielle Mary-Krause, Marialuisa Partisani, Frédéric Bidegain, Laurent Cotte, Elisabeth Aslangul, Antoine Chéret, Franck Boccara, Jean-Luc Meynard, Christian Pradier, Pierre-Marie Roger, Pierre Tattevin, Dominique Costagliola, Jean-Michel Molina, French Hospital Database on HIV

**Affiliations:** 1 Sorbonne Universités, UPMC Univ Paris 06, INSERM, Institut Pierre Louis d’Epidémiologie et de Santé Publique (IPLESP UMRS 1136), F75013, Paris, France; 2 Hôpitaux Universitaires de Strasbourg, Le Trait D’Union, centre de soins de l’infection par le VIH, Strasbourg, France; 3 APHP, Hôpital Avicenne, service de maladie infectieuse, Bobigny, France; 4 Hospices Civils de Lyon, Hôpital de la Croix Rousse, service des maladies infectieuses et tropicales, Lyon, France; 5 INSERM U1052, Lyon, France; 6 APHP, Hôtel Dieu, service de médecine interne, Paris, France; 7 Université Paris Descartes, Sorbonne Paris Cité, Paris, France; 8 APHP, Hôtel Necker, laboratoire de virologie, Paris, France; 9 Université Paris Descartes, Sorbonne Paris Cité, EA 3620, Paris, France; 10 Centre hospitalier de Tourcoing, service des maladies infectieuses, Tourcoing, France; 11 APHP, Hôpital Saint-Antoine, service de cardiologie, Paris, France; 12 Sorbonne Universités, UPMC Univ Paris 06, INSERM (UMRS 938), F75012, Paris, France; 13 APHP, Hôpital Saint-Antoine, service des maladies infectieuses et tropicales, Paris, France; 14 Centre hospitalier universitaire de Nice, groupe hospitalier l’Archet, service de santé publique, Nice, France; 15 Centre hospitalier universitaire de Nice, groupe hospitalier l’Archet, service d’infectiologie, Nice, France; 16 Université de Nice, Sophia-Antipolis, France; 17 Hôpital Universitaire Pontchaillou, service des maladies infectieuses et USI, Rennes, France; 18 APHP, Hôpital Saint-Louis, service des maladies infectieuses et tropicales, Paris, France; 19 Université de Paris Diderot Paris 7, Sorbonne Paris Cité, INSERM (U941), Paris, France; University of Cincinnati College of Medicine, UNITED STATES

## Abstract

**Background:**

The effect of statins on all-cause mortality in the general population has been estimated as 0.86 (95%CI 0.79-0.94) for primary prevention. Reported values in HIV-infected individuals have been discordant. We assessed the impact of statin-based primary prevention on all-cause mortality among HIV-infected individuals.

**Methods:**

Patients were selected among controls from a multicentre nested case-control study on the risk of myocardial infarction. Patients with prior cardiovascular or cerebrovascular disorders were not eligible. Potential confounders, including variables that were associated either with statin use and/or death occurrence and statin use were evaluated within the last 3 months prior to inclusion in the case-control study. Using an intention to continue approach, multiple imputation of missing data, Cox’s proportional hazard models or propensity based weighting, the impact of statins on the 7-year all-cause mortality was evaluated.

**Results:**

Among 1,776 HIV-infected individuals, 138 (8%) were statins users. During a median follow-up of 53 months, 76 deaths occurred, including 6 in statin users. Statin users had more cardiovascular risk factors and a lower CD4 T cell nadir than statin non-users. In univariable analysis, the death rate was higher in statins users (11% vs 7%, HR 1.22, 95%CI 0.53-2.82). The confounders accounted for were age, HIV transmission group, current CD4 T cell count, haemoglobin level, body mass index, smoking status, anti-HCV antibodies positivity, HBs antigen positivity, diabetes and hypertension. In the Cox multivariable model the estimated hazard ratio of statin on all-cause mortality was estimated as 0.86 (95%CI 0.34-2.19) and it was 0.83 (95%CI 0.51-1.35) using inverse probability treatment weights.

**Conclusion:**

The impact of statin for primary prevention appears similar in HIV-infected individuals and in the general population.

## Introduction

Human immunodeficiency virus (HIV) infection induces chronic inflammation and immune activation, even during effective antiretroviral therapy (ART) [1]. Some markers of inflammation are associated with adverse cardiovascular outcomes in both HIV-infected and uninfected populations [2,3]. Statins are widely used for primary prevention of atherosclerotic cardiovascular disease and are known to decrease cholesterol level as well as inflammation. In the JUPITER trial [4], involving apparently healthy men aged 50 years or more and women aged 60 years or more without hyperlipidaemia but with elevated high-sensitivity C-reactive protein (CRP) levels, rosuvastatin significantly reduced all-cause mortality (hazard ratio [HR] 0.80, 95% confidence interval [CI] 0.67–0.97), and the most recently published meta-analysis unequivocally supports statin-based primary prevention [5], with an odds ratio (OR) of 0.86 (95%CI 0.79–0.94) [5]. The effect of statins use on overall mortality in HIV-infected individuals remains controversial [6–9], probably because potential confounders were not adequately taken into account in some studies. In a study a far more potent effect has even been reported in HIV-infected individuals [6] than in the general population [5] without discussion about why. We therefore examined the effect of statin-based primary prevention on all-cause mortality in HIV-infected individuals, by comparison with the general population without prior myocardial infarction or stroke.

### Study population and methods

The patients selected for this study were the controls from a case-control study of myocardial infarction (MI) [10], nested within the French Hospital Database on HIV (FHDH-ANRS CO4), an ongoing, prospective, observational, nationwide, hospital-based cohort of HIV-infected individuals [11]. Enrolments took place from January 2000 through December 2009. Patients with prior cardiovascular or cerebrovascular disorders were not eligible. The outcome measure was all-cause mortality, censored at 7 years of follow-up. Individuals were considered to be statin users if a statin had been prescribed within 3 months prior to the index date, defined as the date of MI in the corresponding case. Patients were followed from the index date until 7 years, or the last follow-up, or death, whichever occurred first. An intention-to-continue approach was used to mimic what would happen in a clinical trial. To include all patients in the analyses, missing values of potential confounders were imputed as previously described [12]. Cases with prior exposure to statin were also corresponding to the definition of statin use for primary prevention, and therefore removing them could lead to a selection bias. However, including all of them would also bias the result by over representing people with MI in our dataset. From January 2000 through December 2009, 96,091 HIV-infected individuals were followed in the FHDH ANRS CO4 cohort. During the same period, 600 HIV-infected individuals had a first validated myocardial infarction, among which 95 patients were on statin therapy, that is 95/96,091; roughly 1/1000. Therefore, to be representative, we should have add around two cases to our study population. In a sensitivity analysis, we randomly selected two of these 95 patients, 10 times, to include them in the analysis. The results of these 10 analyses were combined using Rubin’s rules [12].

The first step of the analysis was to select, among the different ways of modelling continuous variables (as continuous variables, after log transformation, or as categorical variables defined by tertiles), the approach yielding the lowest value of Akaike’s univariable information criterion in Cox models of the risk of death (outcome variable). Then, potential confounders linked either to the risk of death (using Cox models) or to statin use (using logistic regression) were selected. The tested variables were age, gender, HIV transmission group, current CD4 and CD8 T cell counts, CD4 T cell nadir, CD4/CD8 T cell ratio, CD4 T cell nadir/CD8 T cell ratio, plasma HIV-1 RNA level, AIDS status, the haemoglobin level, body mass index (BMI), smoking status, hypertension or use of antihypertensive treatment, diabetes or use of antidiabetic treatment, anti-HCV antibodies and HBs antigen status, non-AIDS malignancy (CIM-10 definition), liver failure, chronic kidney disease, cirrhosis, and pulmonary embolism. For parameters that changed over time, the latest measurement available within 3 months prior to the index date was used. Cholesterol level, which is influenced by statin use, was not considered as a potential confounder but as a consequence of exposure, which could have started many years before, and therefore, as recommended, no adjustment was made on cholesterol level. Variables linked to the risk of death and/or to the risk of statin use were then entered in two stepwise multivariable models, one on the risk of death and the other on statin use, and variables with p values below 0.10 in at least one of the models were retained. Finally, to examine the association of statin use with the occurrence of death, a Cox regression model was constructed that included statin use (yes/no) and the variables retained in the previous steps. The analysis was then repeated using inverse probability treatment weights (IPTW). The propensity score for each subject, defined as the conditional probability of receiving statin therapy given in view of his/her individual covariates, was estimated from a logistic regression analysis that included the same variables as those retained for the Cox model. In the remaining analyses, patients were weighted by the inverse of their probability of statin treatment, using stabilized weights [13].

Sensitivity analyses were used to assess the robustness of the results, by including in the models variables that were not selected as confounders but were known to be linked to the risk of death, such as gender, the plasma HIV-1 RNA level, and prior non-AIDS malignancies.

## Results

Among 1,776 HIV-infected individuals included in the study, 138 (8%) were statin users. The use of statins rose from 3% in 2000–2001 to 13% in 2008–2009. The most frequently prescribed statins were pravastatin (56%), rosuvastatin (32%) and atorvastatin (8%). The characteristics of statin users and non users were presented in Table 1. Patients who were prescribed statins were older (p<0.0001), more likely to have hypertension (p< 0.0001), diabetes (p< 0.0001) and chronic renal failure (p< 0.0001), and more likely to be men who had sex with men (p = 0.0029). They were less likely to have HCV coinfection (p = 0.0025), had a lower CD4 T cell nadir (p = 0.0155), and were more likely to have viral load below 50 copies/mL (p = 0.0004). Current smokers accounted for 38% of statin users and 43% of non users (p = 0.1341). Statin users were less likely to have a BMI below 21 kg/m^2^ (p = 0.0548).

**Table 1:** Characteristics of the study patients.

	Statins users n = 138	Statins non-users n = 1638	P value of univariate conditional logistic regression
Sex, male	125 (90.6)	1454 (88.8)	<0.0001
Age, years	53 ± 10	48 ± 10	<0.0001
Follow-up time, months	48.8 (30.1–73.4)	54.4 (30.8–84.0)	-
HIV transmission group, MSM	80 (58.0)	734 (44.8)	0.0029
**Risk factors for cardiovascular disease**			
BMI			
BMI < 21 kg/m^2^	24 (17.7)	430 (26.3)	
21 kg/m^2^ ≤ BMI < 27 kg/m^2^	95 (68.7)	972 (59.3)	0.0548
BMI ≥ 27 kg/m^2^	19 (13.6)	236 (14.4)	
Smoking			
Current smoker, yes (missing for 17 statins users & 173 non-users)	48 (38.3)	699 (42.7)	
Smoking cessation < 3 years	3 (2.3)	91 (5.5)	0.1341*
Smoking cessation ≥ 3 years	14 (10.5)	142 (8.7)	
Hypertension or antihypertensive treatment, yes (missing for 6 non-users)	41 (29.7)	203 (12.4)	<0.0001*
Diabetes or antidiabetic treatment, yes	30 (21.7)	149 (9.1)	<0.0001
Pulmonary embolism, yes	1 (0.7)	16 (1.0)	0.7702
Hepatic insufficiency, yes	1 (0.7)	16 (1.0)	0.7702
Chronic renal failure, yes	5 (3.6)	7 (0.4)	<0.0001
Cirrhosis, yes	1 (0.7)	28 (1.7)	0.3807
Non AIDS malignancy, yes	6 (4.4)	62 (3.8)	0.7408
Haemoglobin rate, g/dL (missing for 30 statins users & 452 non-users)	14.5 (13.3–15.5)	14.3 (13.3–15.1)	0.4428
Anti-HCV antibodies, yes (missing for 10 statins users & 88 non-users)	6 (4.5)	260 (15.9)	0.0025*
HBs antigen, yes	9 (6.3)	127 (7.8)	0.5540
Characteristics of HIV infection			
Plasma HIV-1 RNA^†^ < 50 copies/mL	73 (52.9)	615 (37.6)	0.0004
CD4 T-cell nadir, cells/mm^3^ (missing for 1 statins user)	139 (46–248)	179 (74–301)	<0.0001*
CD4 T-cell count ^†^, cells/mm^3^	491 (358–656)	466 (312–656)	0.2316
CD8 T-cell count ^†^, cells/mm^3^ (missing for 2 statins users & 29 non-users)	900 (368–1194)	895 (620–1225)	0.9894
CD4/CD8 T-cell ratio ^†^ (missing for 3 statins users & 29 non-users)	0.6 (0.4–0.8)	0.5 (0.3–0.8)	0.1012
AIDS prior to the index date	49 (35.5)	476 (29.1)	0.1109

Table entries are n (%), mean ± standard deviation, or median (interquartile range), as appropriate.

Index date: date of MI diagnosis.

MSM: men having sex with men, BMI: body mass index, AIDS: acquired immune deficiency syndrome, HCV: hepatitis C virus, HBs: hepatitis B surface.

* p values were calculated including missing data.

^†^ Within three months prior to the index date.

**Fig 1:**
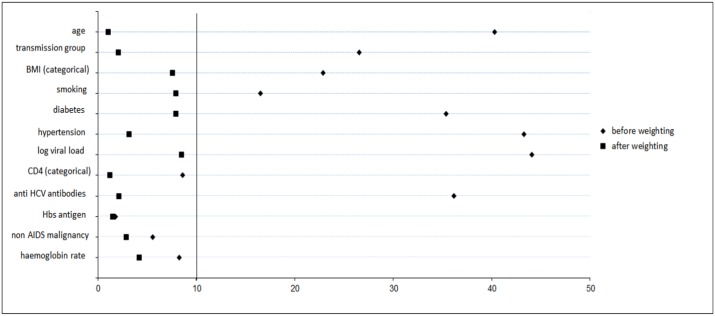
Standardized difference between statin users and non users. Abbreviations: BMI = body mass index, HCV = hepatitis C virus, HBs = hepatitis B, AIDS = acquired immune deficiency syndrome.

Seventy-six deaths occurred during a median follow-up of 53 months, including 6 deaths among statin users. The Kaplan-Meier estimate of 7-year mortality was slightly higher, but not significantly, among statin users than among non users (11% versus 7%, Log Rank p = 0.6335). The following confounders were retained: age (per 10-year increment), HIV transmission group (men who had sex with men versus all other patients), current CD4 T cell count (after log_2_ transformation), haemoglobin level (after log_2_ transformation), BMI (<21, 21–26, ≥27 kg/m^2^), smoking status (never, ceased ≤3 years, ceased >3 years, current), anti-HCV antibodies positivity (yes/no), HBs antigen positivity (yes/no), diabetes (yes/no) and hypertension (yes/no). After weighting and stabilization, the standardized differences between statin users and non users were less than 10% for all the above confounders showing that all non-confounders were well balanced in weighed analyses (Fig 1).

**Fig 2:**
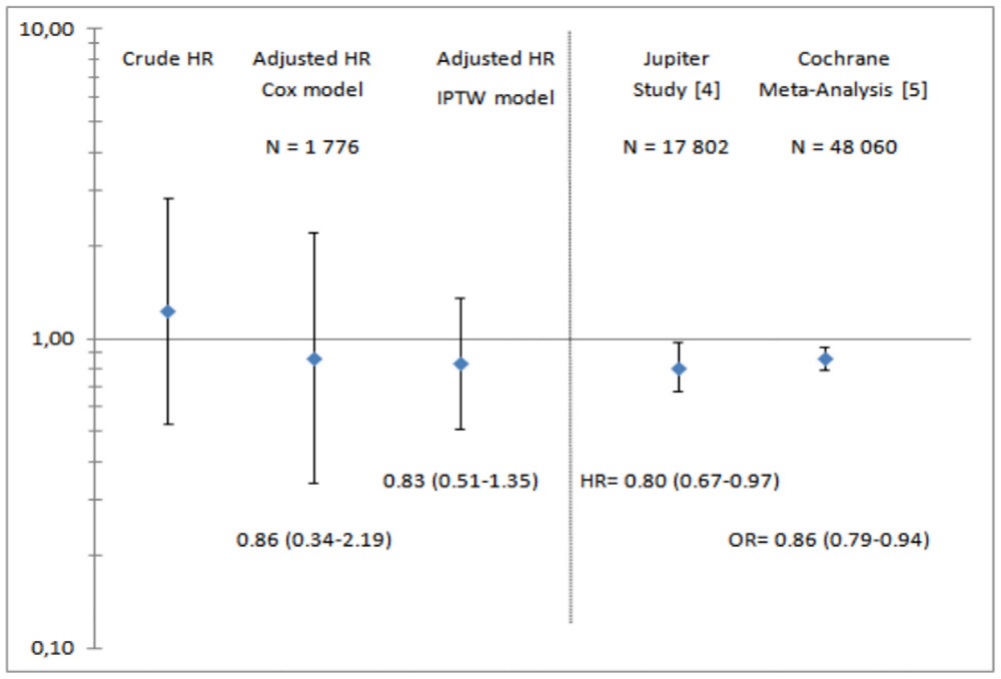
Association between statin use and the risk of death among HIV-infected individuals. Adjustment for gender, HIV transmission group, age, CD4 T cell count, plasma HIV-1 RNA, anti-HCV antibodies and Hbs antigen status, BMI, haemoglobin, smoking, hypertension and diabetes. Abbreviations: HR = hazard ratio, IPTW = inverse probability of treatment weighting.

Association of statin use with the risk of death is depicted in Fig 2. In the univariable model, statin use was associated with an increased risk of death (crude hazard ratio [HR] 1.22), but the association did not reach statistical significance (95%CI 0.53–2.82). After accounting for confounders in the adjusted Cox model, the HR fell to 0.86 (95%CI 0.34–2.19). A similar result was obtained with IPTW (HR 0.83, 95%CI 0.51–1.35). In the sensitivity analysis including two IDM cases receiving statin together with the 1,776 controls ten times, the combined HR was estimated as 0.86 (95%CI 0.66–1.13). Similar results were also obtained in the sensitivity analysis including in the model variables that were not selected as confounders but were known to be linked to the risk of death.

## Discussion

Although the risk of death was higher among statin users than non users in univariable analysis, with a hazard ratio of 1.22 (95%CI = 0.53–2.82), the estimated HR in the adjusted analysis was 0.86 (95%CI = 0.34–2.19), a value similar to that of the general population in the Cochrane meta-analysis [5].

In our study, statins had been prescribed to individuals with an elevated risk of cardiovascular disease and death as illustrated by a higher prevalence of diabetes, hypertension and smoking. Statin use was defined at a fixed time point, the index date. This is a limitation of our study. However, in a setting of free healthcare cost, statin prescription is unlikely to be interrupted in patients at risk. In univariable analysis one would expect to find a higher risk of death among patients treated with statins. When this is not the case [7,8], it points to possible differences in medical coverage or socio-economic status between statin users and non users, and these factors should be taken into account in the analyses. It is also important to take all other confounders into account, while variables that might be affected by statin use, such as cholesterol values on statin therapy, should not. Our results are similar to those of Rasmussen et al. [9] and Overton et al. [8]. In contrast, Drechsler et al. [7] found no effect, with a HR of 0.96 (95%CI = 0.92–0.98), while Moore et al. [6] found a very strong effect with a relative hazard of 0.33 (95%CI = 0.14–0.76). However, Drechsler et al. did not found an increased risk of death in univariable analysis, suggesting difference in access to care among statin users and non-users and they adjusted for the updated LDL-cholesterol level in the multivariable models [7] contrary to the fact that one should not adjust on the consequence of treatment when evaluating treatment effect in an observational setting. Moore et al. did not take into account healthcare coverage or other important confounders such as smoking, hypertension and diabetes. Moore's results were “to good to be true” suggesting remaining confounding, and therefore unlikely to reflect a causal relationship [14]. Although our findings, like those of Rasmussen and Overton [8,9], are more likely to estimate the true effect of statin use on the risk of death among HIV-infected individuals, an effect similar to that in the general population, all three studies lacked sufficient power to show a statistically significant effect.

## Conclusion

The effect of statins on all-cause mortality appears to be similar in HIV-infected and-uninfected populations. The potential benefits of statin therapy in HIV-infected patients should be weighed up in view of the risk of drug-drug interactions and known adverse effects such as the increased risk of diabetes and myopathy [15].
